# Design, challenge, and promise of stimuli-responsive nanoantibiotics

**DOI:** 10.1186/s40580-016-0085-7

**Published:** 2016-10-15

**Authors:** Julius A. Edson, Young Jik Kwon

**Affiliations:** 1 Department of Chemical Engineering and Material Science, University of California, Irvine, Irvine, CA USA; 2 Department of Pharmaceutical Sciences, University of California, Irvine, Irvine, CA USA; 3 Department of Biomedical Engineering, University of California, Irvine, Irvine, CA USA; 4 Department of Molecular Biology and Biochemistry, University of California, Irvine, Irvine, CA USA; 5 132 Sprague Hall, Irvine, CA USA

**Keywords:** Stimuli-responsive, Nanoantibiotics, Drug-resistance

## Abstract

Over the past few years, there have been calls for novel antimicrobials to combat the rise of drug-resistant bacteria. While some promising new discoveries have met this call, it is not nearly enough. The major problem is that although these new promising antimicrobials serve as a short-term solution, they lack the potential to provide a long-term solution. The conventional method of creating new antibiotics relies heavily on the discovery of an antimicrobial compound from another microbe. This paradigm of development is flawed due to the fact that microbes can easily transfer a resistant mechanism if faced with an environmental pressure. Furthermore, there has been some evidence to indicate that the environment of the microbe can provide a hint as to their virulence. Because of this, the use of materials with antimicrobial properties has been garnering interest. Nanoantibiotics, (nAbts), provide a new way to circumvent the current paradigm of antimicrobial discovery and presents a novel mechanism of attack not found in microbes yet; which may lead to a longer-term solution against drug-resistance formation. This allows for environment-specific activation and efficacy of the nAbts but may also open up and create new design methods for various applications. These nAbts provide promise, but there is still ample work to be done in their development. This review looks at possible ways of improving and optimizing nAbts by making them stimuli-responsive, then consider the challenges ahead, and industrial applications.

**Graphical abstract:**
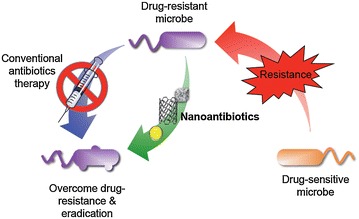
A graphic detailing how the current paradigm of antibiotic discovery can be circumvented by the use of nanoantibiotics

A graphic detailing how the current paradigm of antibiotic discovery can be circumvented by the use of nanoantibiotics

## Background

Since the advent of penicillin, civilization has been in a constant race against microbes that can often evolve in ways that render antimicrobials useless. Bacteria mutate at accelerated rates and readily transfer drug-resistant genes. The development of novel antimicrobials has fallen well behind the rate of mutation. Thus, a need for novel therapeutics is apparent. The scientific community has been taking various approaches to address the problem from discovering new drugs such as teixobactin [1] and its synthetic forms [2], to repurposing old antibiotics through use of nanoparticles [3, 4]. Another proposed method of addressing the bacterial rate of drug-resistant formation is using RNAi and antisense technology to remove the silencing trait to sensitize the microbe [5–9]. One thing that is clear is that there is a desperate need for innovative therapeutics.

**Fig. 1:**
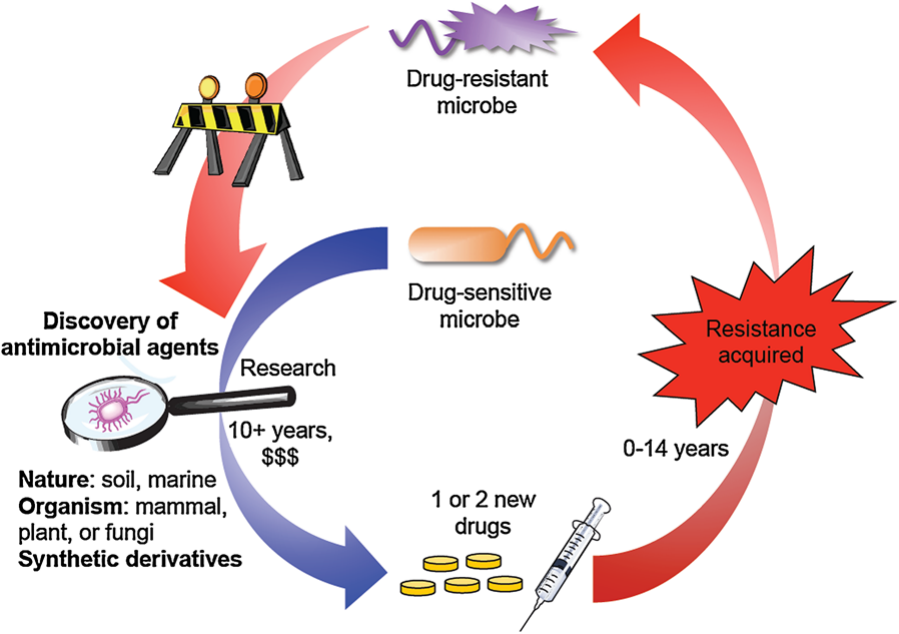
Current drug discovery paradigm in antibiotics. Scheme of the current paradigm in drug discovery. A target microbe that requires new antibiotics becomes the main subject of research. The discovery of new antimicrobial agents is usually discovered in soil or marine bacteria, and synthetic derivatives are generated. Based on current trends, these have up to 14 years of efficacy before resistance is developed. The resistance is usually acquired through horizontal gene transfer

The problem, is that while all these new antimicrobials are effective and promising, they remain as only short-term solutions to the overall challenge of drug-resistant microbes. It takes anywhere from of a few months to approximately ten years for a resistant gene to develop [10]. Aside from environmental pressure and overuse, another cause for this rapid loss of efficacy is due to the fact that most available antibiotics are derived from a compound discovered in a microbe for an example, penicillin, Fig. 1. While the source microbe is resistant against the compound [11, 12], the unfortunate reality is that the resistant gene can be easily passed on through horizontal gene transfer [5, 11] to a target microbe. For us to truly gain an advantage against microbes, we need to engineer and design methods to avoid that cycle.

Recently, the use of nanoantibiotics as a therapeutic strategy has gained a lot of attention [10, 13, 14]. Nanoantibiotics (nAbts) are nanomaterials that have an antimicrobial activity or improve the efficacy and safety of antibiotics administration [10]. nAbts possess many advantages over conventional antibiotics, including but not limited to production, storage, durability, and versatility. Preparation of antimicrobial nanoparticles may be cheaper, faster, and more adaptable with the added advantage of a long shelf life [10, 15]. They are typically composed of either naturally occurring antibacterial substances, metals and metal oxides, carbon-based nanomaterials, or nanoemulsions [3, 14]. Moreover, the persistent misuse and overuse of current antimicrobials can also be attributed to the lack of patient compliance [16–18]. nAbts provide the added advantage of a site-specific, sustained release that can be administered in a single dose. While most conventional antibiotics are a systematic release of therapeutics that require multiple doses.

**Fig. 2:**
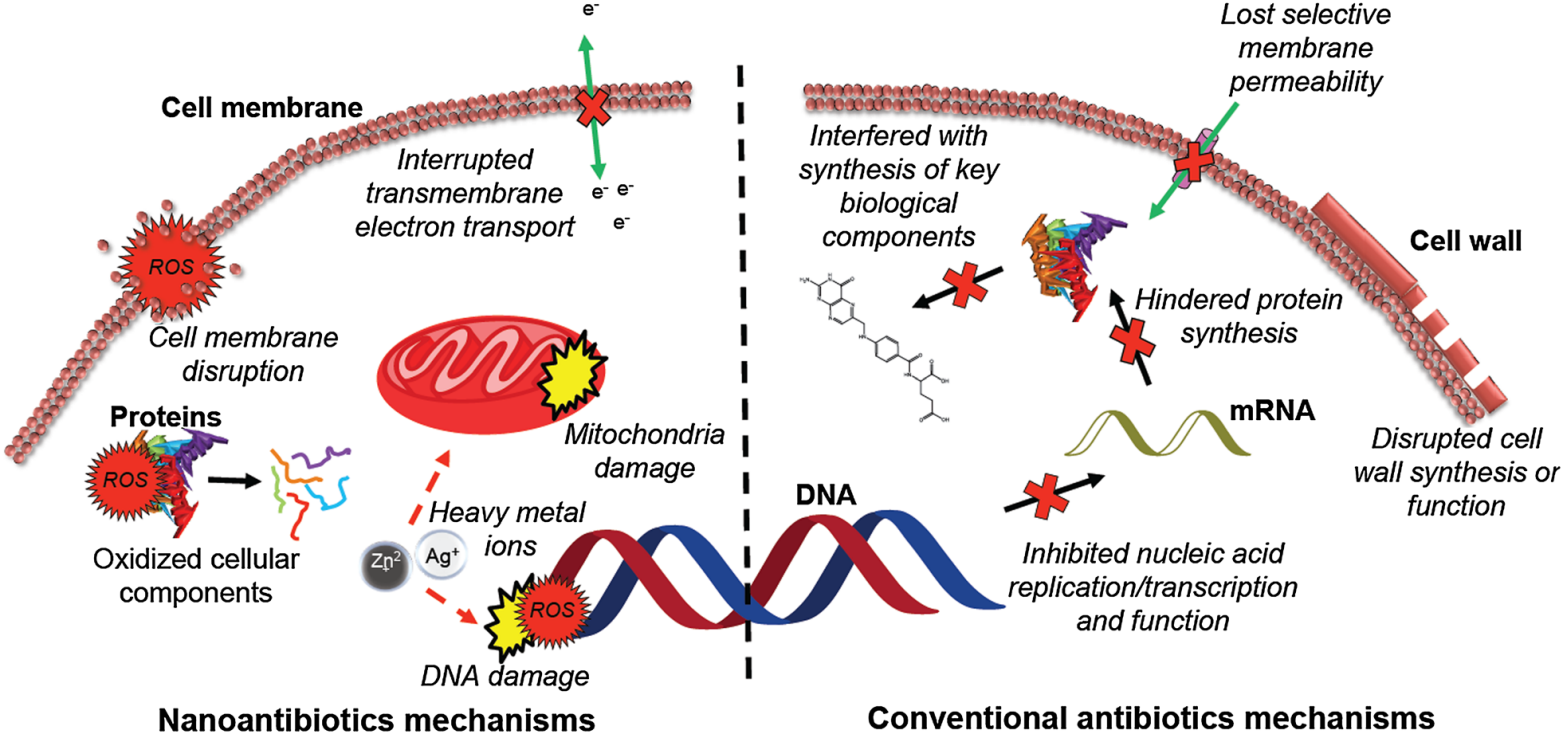
Nanoantibiotics mechanisms versus conventional antibiotic mechanism. A schematic illustration of nanoantibiotics (nAbts) mechanism in comparison to conventional antibiotics mechanisms. nAbts mechanisms are typically cell membrane disruption, and oxidation of cellular components caused by reactive oxygen species (ROS); interruption of transmembrane electron transport; and mitochondria and DNA damage caused by heavy metal ions and ROS. Conventional antibiotics mechanism inhibits nucleic acid transcription and function caused by quinolones, fluoroquinolones and rifamycins. Additionally, other conventional antibiotics can hinder protein synthesis, disrupt cell all synthesis or function, cause a loss of selective membrane permeability, or interfere with the synthesis of key biological components such as folic acid

The improved antimicrobial efficacy can be attributed to high surface area to volume ratios and unique chemical–physical properties. Some proposed antimicrobial mechanisms of nAbts include: (1) generation of reactive oxygen species (ROS) that age bacterial intracellular components, (2) compromise the bacterial cell wall/membrane, (3) interruption of energy transduction, and (4) inhibition of enzyme activity and DNA synthesis [10, 15], as shown in Fig. 2. In comparison, conventional antibiotic agents have treated a diverse multitude of microbes via interference with the integrity of the cell wall or membrane, hindrance DNA or RNA synthesis, or disruption of protein or amino acid synthesis, Fig. 2 [5]. While there is some overlap present, such as inhibition of nucleic acid synthesis and compromising cell wall integrity, the mechanisms by which these occur are vastly different. While conventional antibiotics interfere on the molecular scale, nAbts physically disrupt key biological processes. This allows for a unique method of destroying microbes with a combination of both mechanisms. This review will provide an overview of various stimuli-responsive linkers used to design a multipurpose nanoantibiotics, the manufacturing of nAbts and the market potential for their production and use.

## Different types of nanoantibiotics and the promise of stimuli-response

NAbts come in different shapes and sizes, all of which have been continuously documented [3, 4, 10, 19, 20]. Briefly below, and in Table 1, is an overview of some of the possible category nAbts along with some brief examples.

**Table 1:** Comparisons of various nanoantibiotics

Class	Example	Pro	Con	Mechanism	Current usage	References
Polymers	Chitosan	Biocompatibility, cationic properties, cost	Insoluble in biological pH	Cell membrane destabilization, enzyme inactivation	Bacterio-static agent, coating for implants, water purification	[21–24]
	Gelatin	Biocompatibility, polymer size uniformity, cost	Preparation, lack of muco-adhesive properties	Destabilization of membrane	Food additive, immunoassay	[25–27]
	Stearyl-melittin	Membrane lysis potential, minimal toxicity	Material preparation	Cell membrane depolarization, inhibition of biopolymer synthesis	Gene transfection	[31]
Metals and metal oxides	Gold	Photo-thermal and optical activity	Non-biodegradability	Cell membrane disruption	Photo-thermal therapy, adjuvant	[34, 35]
	Silver	Multi-microbe efficacy	Toxicity	Release of heavy metal ions, multiple effects	Coatings, wound dressing, filters	[4, 10, 36]
	Titanium dioxide	Magnetic and photocatalytic activity	Ease of clearance	ROS generation, damage cell wall and membrane	Food, purifiers, water treatment	[5, 37]
Carbons	Fullerenes	Site-specificity in vivo	Acute toxicity	Electron transport disruption	Disinfectants	[39]
	Nanotubes	Manufacturing ease, photo-thermal and photodynamic activity	Toxicity	Cell membrane disruption by ROS, intracellular component oxidation	Water filtration, coatings, antifouling membranes, wound treatment	[3, 40, 41]

### Polymers

Nanoantibiotic polymers typically need to be formulated into nanoparticles for full usage of the therapeutic antimicrobial properties. These polymers do however hinder the growth of bacteria through one of the nAbts mechanisms. While there are most likely many others that are not identified, examples of some well-known polymers are listed. Chitosan is a partially deacetylated chitin that has been shown to possess a wide spectrum of antibacterial activity [21, 22]. Chitosan’s antimicrobial mechanisms include increasing the permeability of the microbial wall or chelation trace metals [23, 24]. Gelatin is derived from type I collagen composed of glycine- and proline-rich repeating units [25–27]. Gelatin has been shown to damage the cell membranes of *Staphylococcus aureus* [26, 27]. An additional class of polymeric materials are peptides, which have been developed as a treatment against various microbes including drug-resistant strains [28–30]. For an example, stearylated melittin, is a well-known cationic antimicrobial peptide, which creates pores in the microbe membrane [31]. Furthermore, some synthetic and natural polymers may be considered as nAbts based on their abilities to act as matrices or vectors for varied deliveries. An example of this is dextran or acetylated dextran. Though dextran has not been shown to demonstrate any innate antimicrobial properties, it is an easily modifiable polymer that can aid in antimicrobial therapy [32, 33].

### Metals and metal oxides

Gold-based nanoparticles have been used to treat bacterial infections using heat generated from photo-thermal effects. Gold’s antimicrobial activity is caused by strong electrostatic attractions to the negative charge bilayer of the cell membrane [34, 35]. Silver, on the other hand, has been used since ancient times as an antimicrobial. Amongst all the different types of metallic nanoparticles, silver NPs have proven to be the most effective against various types of microorganisms [10]. Silver NPs work by interfering with the respiratory pathway and cell division. Additionally, the release of silver ions further enhances antibacterial activity [4, 10, 36]. Titanium dioxide (TiO_2_) NPs are also well known for photocatalytic antimicrobial activity [5, 37] as demonstrated by the strong bactericidal activity of TiO_2_ upon receiving irradiation with near-UV light and UV-A. TiO_2_ NPs work by producing ROS such as free hydroxyl radicals and peroxide. Finally, zinc oxide NPs are nontoxic and biocompatible metal oxide NPs that have been widely adopted throughout the biotechnology and consumer goods industries [3, 38]. They are antibacterial against some important foodborne pathogens [3, 10], disrupt lipids and proteins of the bacterial cell membrane, and promote the generation of hydrogen peroxide and Zn^+2^ ions [10].

### Carbon-derived NPs

Fullerenes—and more specifically, colloidal C_60_ aggregates (nC_60_) in water—have been recently discovered to have beneficial antimicrobial properties [39]. Suggestions for the antibacterial mechanism for nC_60_ includes photocatalytic ROS production and lipid peroxidation in the cell membrane [10]. Other modifications of fullerenes allow for the destruction of microbes by insertion into the cell wall, which is then followed by the disruption of the cell membrane structure [3]. Additionally, single-walled carbon nanotubes (SWCNTs) and multi-walled carbon nanotubes (MWCNTs) have been used for a multitude of antimicrobial applications [40, 41]. CNTs destroy gram positive and gram negative bacteria through irradiation mediated oxidative stress which affects the membrane integrity and metabolic activity of the bacteria [3, 42].

### Promise of stimuli-response

**Fig. 3:**
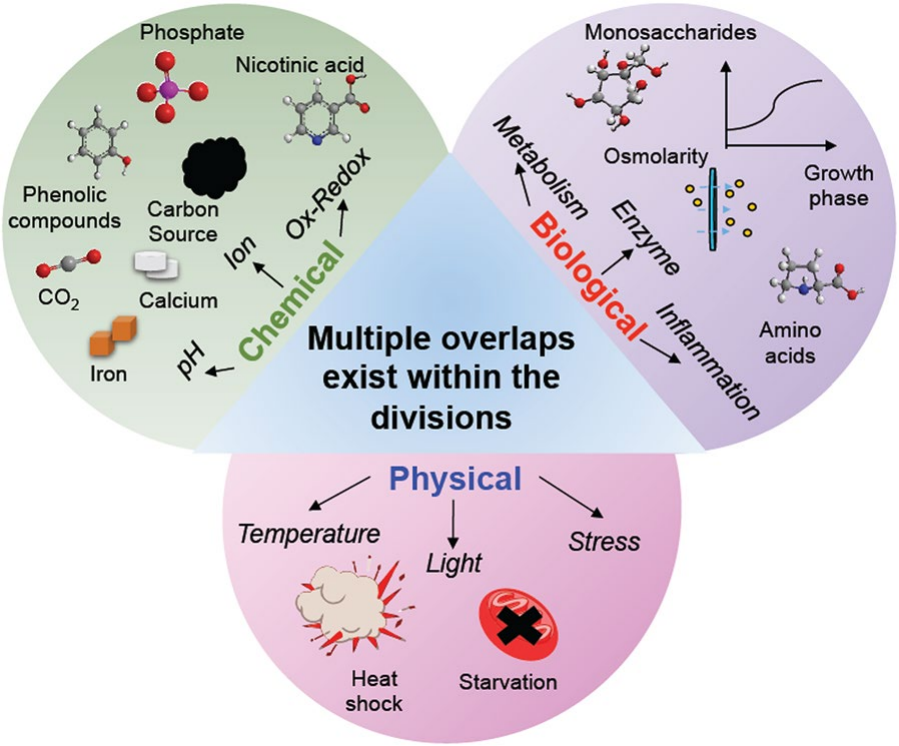
Environmental regulator of virulent factors matched with stimuli classification. A schematic diagram of virulent factors that microbes possess which can be regulated by environmental signals which can be classified as chemical, biological, or physical

In addition to using stimuli-responsive linkers to combine conventional antibiotics and nAbts, these linkers are able to provide a unique advantage over current antibiotic strategies: they can be effectively tuned to certain infections or sites. To note, all organisms exist with a specific microenvironment around them. These environments are dynamic and deeply influenced by the organisms within them. Such phenomena can be observed in bacterial colonies, both commensal and pathogenic [43]. In the case of pathogens, environmental cues usually determine the virulence factor. The virulence factor of an infection is an intrinsic trait that is usually measured in terms of morbidity or mortality [44]. For most microbial infections, these virulent factors are responsible for efficient multiplication in the host with such attributes as adherence to host tissues, production of host-specific toxins, invasion into host cells, and resistance to the host defense mechanisms. Furthermore, a large proportion of these virulence factors have specific environmental signals such as temperature, osmolarity, pH, oxygen, carbon dioxide, amino acids, and iron levels, as demonstrated in Fig. 3. Pathogens may use these factors as signals to detect their environment, for instance, cueing them as to whether they are in the gut versus the lung or intracellular versus extracellular environments [44]. By taking advantage of these environmental cues, highly effective and multi-purpose nAbts can be created.

## Stimuli-responsive linkers and combinatory therapeutic design

It is clear that there is a synergistic opportunity in designing nanoparticles with a stimuli-responsive linker. These stimuli can either be physical, chemical, or biological [45]. For this review, we will focus on stimuli responses that can be easily added to a nAbts system and allow for improved efficacy in certain environments. These stimuli of interests are pH, temperature, redox, enzymes, and light.

### pH-responsive

pH-responsive linkers have a unique advantage against microbes that thrive in highly acidic environments, whether extracellularly in the stomach (pH = 1–3) and gastrointestinal tract (pH = 5–8) or intracellular in the phagolysosomes (pH = 4.5–5) and macrophages [45–47]. Additionally, chronic infections and wounds have pH values between 5.4 and 7.4 and are another potential site for the application of this therapy [45]. Examples of microbes that thrive in an acidic environment include *Helicobacter pylori*, *Agrobacterium tumefaciens* or *Vibrio cholera* [45, 46]. This specificity allows for a direct exploitation of the stimuli-response in certain tissues or in a cellular compartment. The key element for pH-responsiveness is protonation/deprotonation caused by charge distribution over ionizable functional groups such as carboxyl or amino groups [46] listed in Table 2. Polymers that contain carboxylic, sulfonic acid or amino groups such as poly(l-histidine), poly(methacrylic acid) (PMAA),and poly(acrylic acid) (PAA) [48], have been commonly used for their pH-responsive functionalities. pH changes induce a phase transition in pH-responsive polymers very abruptly, which can aid within intracellular compartments or with rapid release of drug cargos [46, 48].

**Table 2:** pH-responsive functionalities

Name	Structure	pH Range	References
Ketal		4–5	[48, 52, 92]
Acetal			[48, 52]
Hydrazone		<5	[48]
Hydrazide			[48]
Oxime			[48]
Methyl maleate		5.5, 6.8	[48]
Succinyl		5–7	[46, 51]
Carboxymethyl			[46, 52]
Imine			[48]
Amino ester			[93]
Acetyl		6–7	[46, 94]
Histidine			[48]
Phthalyl			[46]

### Temperature-responsive

Changes in an organism’s temperature have been correlated with dramatic changes in the expression of virulent factors. Additionally, multitudes of diseases have been known to manifest temperature changes [44, 45]. For example, *Salmonella typhimurium, Bordetella pertussis, Escherichia coli, Vibrio cholera, and Shigella* sp. have been shown to rely on temperature dependent environmental signals [44]. Normally, a temperature-dependent response is characterized by a critical solution temperature where the hydrophobic and hydrophilic interactions abruptly change within a small temperature range [45, 49]. This change induces the disruption of intra- and intermolecular electrostatic and hydrophobic interactions and results in collapse or expansion (a volume phase transition) [45, 47, 50]. Materials containing this group either become insoluble upon heating, classed as lower critical solution temperature (LCST), or become soluble upon heating, classed as upper critical solution temperature (UCST) [50]. LCST materials, Table 3, are usually based on N-isopropylacrylamide (NIPAM), N-vinylcaprolactam (NVCl), methylvinylether (MVE) [45, 47, 50–52]. Whereas typical UCST systems are based on a combination of acrylamide (AAm) and acrylic acid (AAc) [47, 50].

**Table 3:** Functionalities responsive to temperature, redox, light, and hypoxia

Name	Structure	Trigger	References
Ethoxyethyl glycidal ether		Heat: Δ @ 29.6–40.4 °C	[50]
NIPAM–acrylamide		Heat: Δ @ 30–32 °C	[47, 48, 50]
Methylvinylether		Heat: Δ @ 35–37 °C	[47, 50]
2-(2-ethoxy) ethoxyethyl vinyl ether		Heat: Δ @ 41 °C	[50]
Disulfide		Glutathione reduction	[49, 52, 56]
2-Nitrophenyl ester		Ultraviolet light >310 nm	[45, 51, 52]
Spiropyran		Light	[51, 52]
Azobenzene		Hypoxia and light	[45, 48, 51, 52]
Nitroaromatic		Hypoxia	[95]
Quinone			[45]

### Light- and redox-responsive

While light might not be an environment dependent stimulus, it is an excellent external stimulus that can be applied to antimicrobial therapy. By combining UV exposure and drug release, light-responsive systems become very advantageous, particularly for antimicrobial systems because light can be applied instantaneously and under specific conditions with high accuracy [45, 48]. The wavelength of the light producing laser is tuned to near-infrared which is less harmful and has deeper penetration in tissues than visible light. Most photo-responsive materials possess light-sensitive functionalities, Table 3, such as azobenzene, spiropyran or nitrobenzyl groups [51, 52]. Examples of light-responsive antimicrobials can be seen in studies using modified TiO_2_ as a photocatalyst against such pathogens as *E. coli* [53, 54]. Additionally, the use of azobenzene- based antimicrobial compound has aided in the creation of polymer films active against *S. aureus* and *C. albicans* [55].

When trying to add redox-response functionality, researchers have typically focused on disulfide groups as these are unstable in a reducing environment [46, 48, 51, 56]. There is a redox potential (~100–1000 fold) that exists between extracellular and intracellular environments. The extracellular space environment is oxidative while intracellular is reductive due to glutathione concentration. Disulfide links degrade when exposed to glutathione or cysteine [52]. This degradation allows researchers to tune for environmental signals. At times, the redox reaction will alter the hydrophobic and the hydrophilic properties of a molecule, leading to a responsive swelling and de-swelling [51, 52] which is another responsive that can be taken advantage of for a nAbts therapy.

### Enzyme-responsive

All organisms create special enzymes to help them thrive in their microenvironments. For example, bacteria in the colon produce reductive enzymes or hydrolytic enzymes capable of degrading various types of polysaccharides [57]. Researchers can take advantage of this particular characteristic since most infection sites will overexpress a certain enzyme, and responsive functionality can be applied to the material of interest [44]. Usually, in enzyme-responsive systems, enzymes are used to destroy the polymer or its assemblies [57]. Enzyme-responsive systems have many advantages, the largest being that they do not require an external trigger for their decomposition, Table 4. They also exhibit high selectivity and work under mild conditions [57]. For example, designing linkers that are responsive to pyroglutamyl-peptidase I [58, 59], a protease found in *S. aureus,* can allow for the creation of a nAbts that would selectively release at that infection site. The major drawback of enzyme-responsive systems, however, is the difficulty in establishing a precise initial response time [57].

**Table 4:** Enzyme-responsive functionalities

Type	Example	Activity	Current application	References
Proteases	Pyroglutamyl-peptidase I (*Staphylococcus aureus*)	Cytosolic hydrolysis of terminal amino groups	Diagnosis, protein sequence analysis, antibody target	[58, 59, 99–80]
Lipases	PAL (*Pseudomonas aeruginosa*)	Hydrolysis of glycerol esters	Synthesis of industrial compounds	[100, 101]
Glycosidases	EndoS (*Streptococcus pyogenes*)	Modulating the IgG effector functions	Immunomodulation, glycan analysis	[102–104]
Urease	UreA, UreB (*Helicobacter pylori*)	Conversion of urea to ammonia, neutralization of pH	Taxonomic identification, vaccine candidate	[105–107]
Glucose oxidase	GOx	Catalysis of glucose oxidation	Food processing, antibacterial, antifungal	[108–111]
Peroxidase	KatG (*Mycobacterium tuberculosis*)	Activates isoniazid, a frontline anti-TB drug	Proteomics, diagnostics	[112–116]
Esterase	ADP1 (*Acinetobacter* sp.)	Carbamate and ester cleavage	Drug abuse treatment, biocatalysis	[117, 118]
Amidase	AmpD (*Citrobacter freundii*)	Amide cleavage, cell wall recycling	Diagnostics	[119–121]

Applying stimuli-responsive functionalities to the design of nAbts opens up the possibility of creating a multipurpose antimicrobial with an environmental dependent release. Additionally, the opportunity to include a targeted drug release functionality is presented. Thus, there is a synergist circumstance where the drug can find renewed efficacy against a drug-resistant organism.

### Combination of drug molecule with nanoantibiotics

While nAbts alone exhibit excellent antimicrobial properties, an even more effective therapy can be engineered by combining the properties of nAbts with that of conventional antibiotics. nAbts can target one mechanism of a microbe whereas conventional antibiotics can be used to address another, thus creating a combinatory therapeutic system that can tune to a number of scenarios. For example, it has been shown that there is a cooperative antimicrobial effect occurs when chitosan is combined with sulfamethoxazole to combat drug-resistant *Pseudomonas aeruginosa* [60]. Additionally, chitosan-capped gold nanoparticles coupled with ampicillin presented a twofold increase in efficacy [61]. A similar trend is observed when various aminoglycosides (streptomycin, gentamycin, neomycin) were conjugated to gold NPs [62]. Silver NPs has also been shown to significantly increase the antibacterial activities of drugs such as ampicillin, kanamycin, erythromycin, and chloramphenicol when tested against *E. coli*, *S. typi*, *S. aureus*, and *M. luteus* [63]. The clinical potential for such combinatory uses of nAbts and small drug molecules against drug-resistant infections, can also be aided by the addition of a stimuli-responsive linker, this is exemplified by the improved efficacy found in such attempts [4, 26, 35, 40, 64].

### Nanoparticles with multi-stimuli response

**Fig. 4:**
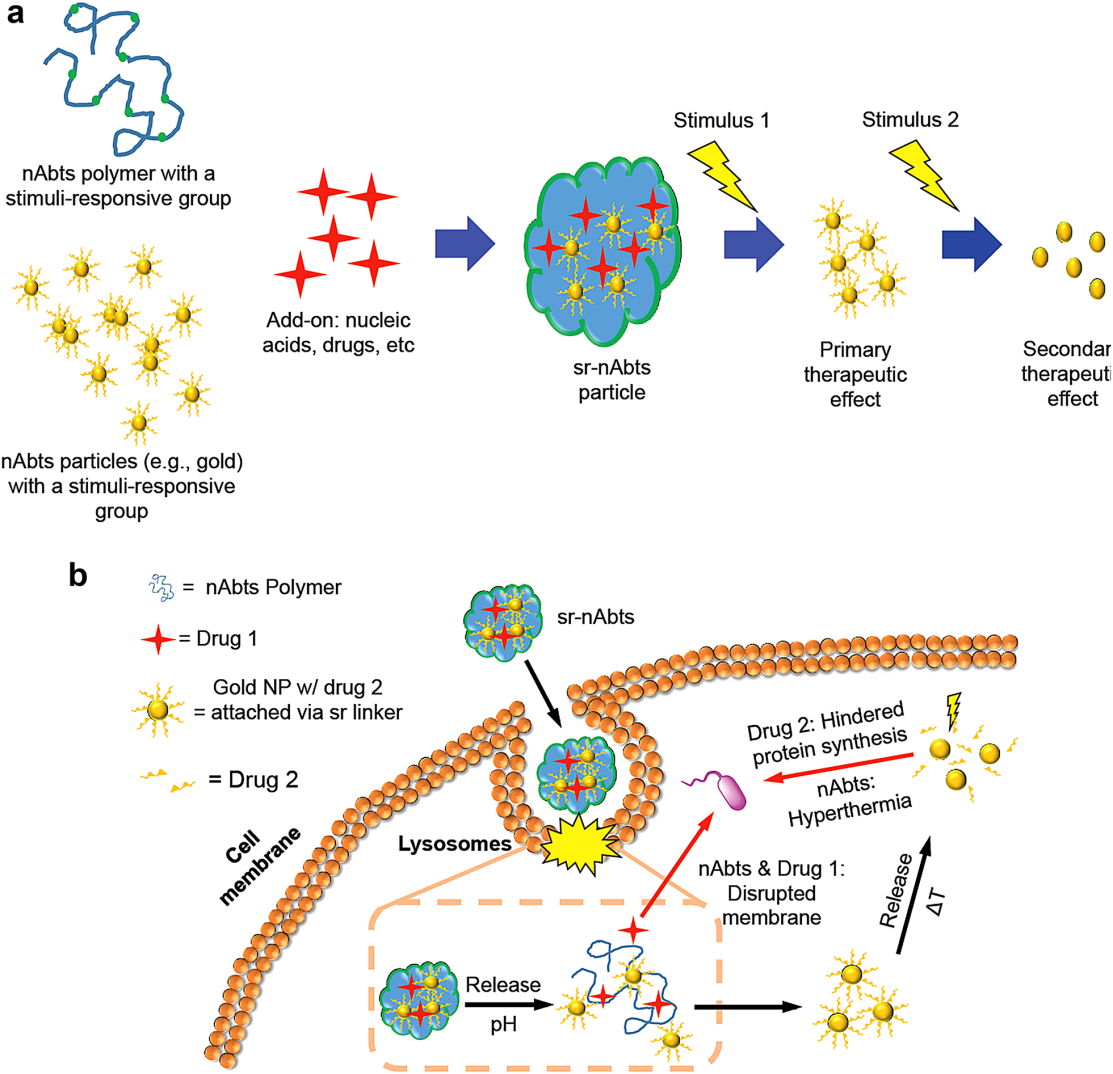
**a** Formation of a stimuli-responsive nanoantibiotics particle: a combined system can be created by combining conventional antibiotics with nanoantibiotics. Stimuli response can aid in the synergistic efficacy by allowing a particle to display multiple therapeutic effect based on the stimulus applied. **b** Proposed therapeutic effect of stimuli-responsive nanoantibiotics against intracellular infection. To target intracellular microbes, conventional antibiotics are combined with nanoantibiotics with stimuli response. Upon endocytosis, these stimuli-responsive nanoantibiotics (sr-nAbts) are triggered to release after certain environmental triggers. With pH triggered release, the particle unpacks to escape the endosome, target the microbe with 1 drug and nAbts. Based on the infection, a second trigger can release another drug and use an additional nAbts mechanism

Another advantageous nAbts design is one that places various stimuli into one package. These will require the use of different stimuli-responsive linkers with two or more nAbts. For instance, a thermo-responsive group like NIPAM is combined with pH-responsive functionality for the purpose of designing a multi-environment NP [47]. These could be very useful in topical applications where one release will happen at body temperature, followed by another stimulus-triggered release intracellularly or at an inflammatory site. Moreover, a multi-stimuli nAbts can be advantageous for treating difficult intracellular drug-resistant microbe as illustrated in Fig. 4. Adding a stimuli-responsive functional group to a nAbts such as a gold nanoparticle or a carbon nanotube can be accomplished by different methods from surface coating using emulsion to reduction-driven synthesis [63, 65]. For polymeric systems, the method that dictates the addition of stimuli-responsive functional group is determined by the specific group being conjugated. For an example, conjugating a pH responsive functionality such as succinyl or acetyl to chitosan has been done by various groups with relative ease [66–68]. An alternative pairing is one that places a light-responsive nanoparticle with one that has a different antimicrobial mechanism. For example, doping TiO_2_ with silver creates a particle that can destroy bacteria by photocatalytic inactivation and generation of silver ions [36, 69, 70]. These particles have been shown to have excellent light-independent antimicrobial activities against *E. coli, S. aureus* and *P. aeruginosa* [69]. A similar interaction can be observed when gold is combined with TiO_2_ NPs. While not necessarily used for antimicrobial therapy yet, various groups in the cancer research field are already applying the strategy of multi-stimuli-responsive particles [71].

## Challenges for nanoantibiotics

With all the promise of nAbts comes challenges that must be addressed before widespread clinical use can be adopted. The two main challenges of nanomaterials in biological applications are toxicity of the material and large scale manufacturing of those materials.

### Toxicity

Various nAbts, particularly metallic and carbon-based ones, have severe toxicity generated from prolonged exposure. Although silver NPs provide many benefits, prolonged exposure to soluble silver-containing compounds may produce an irreversible pigmentation in the skin (argyria) and the eyes (argyrosis) in addition to other toxic effects [10]. Some studies provide a direct contradiction to this, claiming minimal concentration dependent toxic effects [10, 72]. These concentration dependent toxicities to mammalian cells have been shown in other studies using metallic nanoparticles [73, 74]. Similarly, while nanotubes and fullerenes have been shown to be potentially toxic, there are some contradictions which suggest that the toxicity may be due to solvent contaminants during preparation [39, 75]. For one, a suspension of nC_60_ prepared without THF lacked toxicity [76]. Moreover, nC_60_ prepared without using any polar organic solvent lacked any acute or subacute toxicity in rodents [77]. And although these potential side-effects limit their applications, their use should not be disregarded entirely. A complete elucidation of nanoparticle toxicity needs to be ascertained before extensive manufacturing induced exposure.

In general, there has been increased scrutiny over the toxicity of nanoparticles due to increased use in various industries. Not only in the application, but also in the manufacturing of nanoparticles as those who manufacture the nanoparticles will experience the most exposure. Silver NPs have been analyzed to determine their toxicity when manufactured. Based on a continuous 3-day exposure assessment, it was evident that workers were exposed to high levels of nanoparticles on a day-to-day basis; similar results were also found for other metallic and carbon NP manufacturing [78]. While the exact toxicities of long-term overexposure are still completely unknown, a recent study by Das et al. [79] looks at the potential toxicity to mammalian germ cells and developing embryos from engineering nanoparticles such as gold, silver, fullerenes, and chitosan. They summarize various toxicities based on nanoparticle uptake and internalization mechanisms. The major emphasis of the study is that while some nanoparticles may not have any acute toxicity, there is a possibility that there is some long-term toxicity or effect to germ lines to consider. The authors disclose that a large percentage of these toxicities are mostly dependent on NP size and surface modifications. Taking those variables into consideration when designing and engineering nAbts can aid in ameliorating the possible toxicities that may arise.

### Large-scale manufacturing

**Fig. 5:**
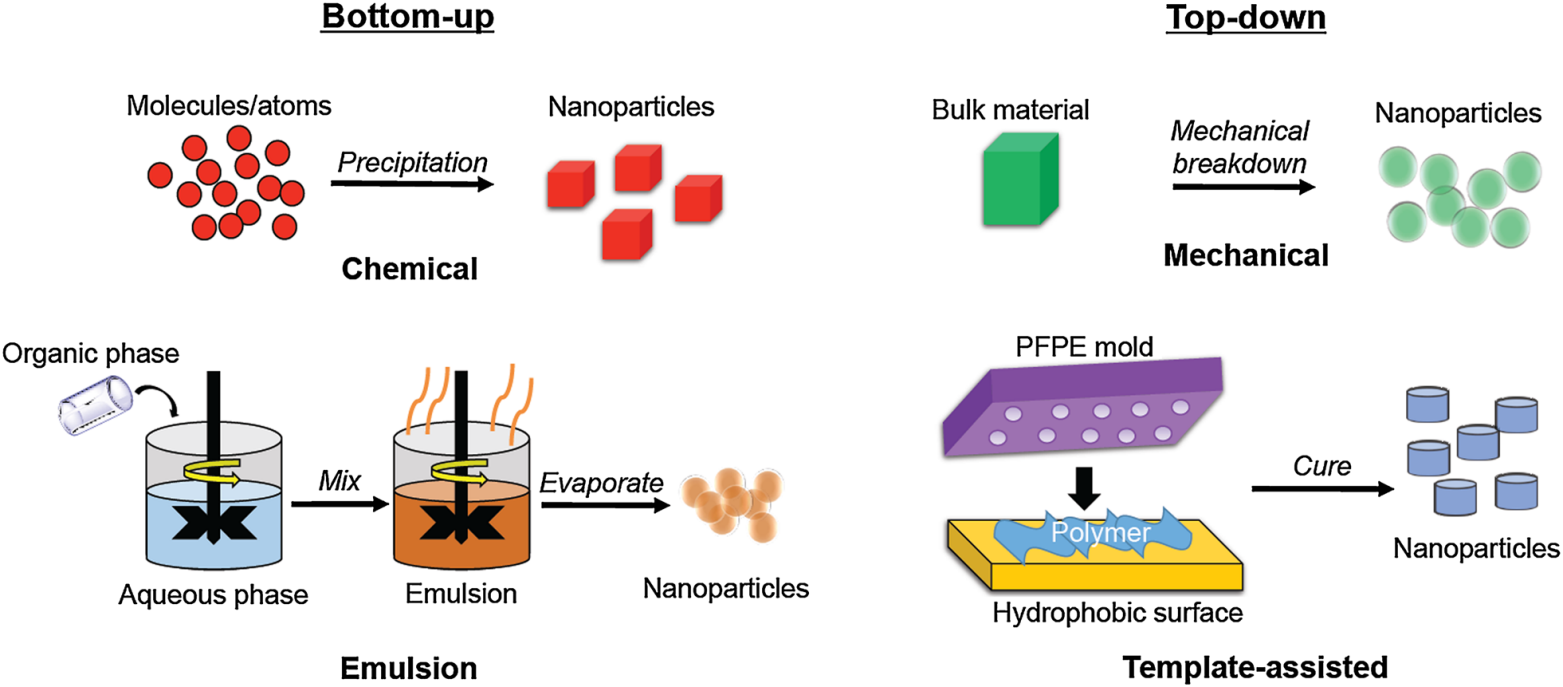
Manufacturing methods of nanoparticle. Nanoparticles can be manufactured in large scales either by bottom-up or top-down manufacturing methods

Scale-up for creating nanoparticles requires sophisticated techniques [80]. Two key pathways to generate nanoparticles are through chemical or mechanical routes [37]; these can also be considered as bottom-up or top-down manufacturing approaches respectively, Fig. 5 [81]. A bulk material may be dissolved chemically into molecular entities to yield a distinct molecular intermediate form of the material. That intermediate is then reacted kinetically or processed further through the use of stabilizing agents such as emulsifiers. Generation of silver nanoparticles from silver nitrate is an excellent example of a chemical route [37]. Alternatively, mechanical energy can also be applied to a bulk material to split it into smaller particles. This usually requires heavy machinery such as a mill. Regardless of the chosen pathway, most particles are modified, whether by some type of surface modification or another type of customization, before further use [37].

Another method used to industrially generate nanoparticles is an emulsion type system [82, 83]. This is a bottom-up synthesis approach that has some similarity to chemical synthesis route. Instead of distinct intermediates being formed, the emulsion route allows for the formation of nanoparticles based on an oil–water system. The versatility of the reactor system, where starting materials may simply be mixed, serves as an advantage for this system. A disadvantage of the emulsion system is that it will be limited to only certain types of nanoparticles made with materials that are soluble in an organic phase or an aqueous layer, and thus, it cannot be widely used.

The other end of the spectrum for scaling and manufacturing is the top-down approach of templated systems [84, 85]. Using cast molding generated from polydimethylsiloxane (PDMS), different shapes and sizes can be generated for a material that can be cured and dried, usually a polymeric material. While most nAbts are not applicable to this technique, there might be a way of combining the stimuli-responsive linkers or polymers that will allow for the templated-assisted system to be leveraged. The main advantage of using a template-like system is the ability to make many uniform nanoparticles very rapidly. This would reduce the cost of manufacturing for pharmaceutical applications while being able to maintain consistent quality. This consistency is something that the bottom-up approach may lose. The downside is the limited source materials that can be used in the template-assisted systems.

One thing is certain, when industrial scale manufacturing of nAbts comes into question, attempting to use just one system may not be adequate enough to serve a broad therapeutic need. Entities that decide to venture into commercializing nAbts for therapeutic applications need to consider which systems they are trying to target, and what would be the safest and most economical way to accomplish that goal.

## Future directions for nanoantibiotics

Worldwide, scientists are scrambling to discover and uncover new ways to fight off infections. The rate of drug-resistant formation is frightening, and while there are many promising new compounds and products being introduced, unfortunately, that is still not enough. The major problem lies in the fact that we are stalled in this persistent paradigm of discovering new antibiotics. Of the few pharmaceutical companies that invest money into the discovery of new antibiotics, most do not see a return on their investment. This makes the prospect of developing new antibiotic highly unattractive from that standpoint.

However, the difficulty in developing new antibiotic compounds stems from the fact that most new antibiotic compounds are found from another, competing microbe [12, 86]. nAbts circumvent that system by using a completely different mechanism of action; moreover, they are also highly versatile and tunable. This allows for the ubiquitous use of nAbts. Antimicrobials are used in a multitude of industries such as livestock and agriculture, water treatment, military, and clinics; however, sr-nAbts will most likely thrive in industries that require the precise release of certain antibiotic effect after certain conditions are met. One possible application is to apply it to on-site or field-based medical devices, where autoclave or sterilization is not easily accessible. This will allow for situations where temperature, light, or pH responsive nAbts coated materials can react to sterilize an environment. The same system can also be used in the design of a water treatment system where the filter self-cleans and sterilizes itself. Another possible direction would be a theranostic system for antimicrobial infections. This is a relatively direct venture if the nAbts system is stimuli-responsive. The timing of such a system would be most useful in topical applications, or applications where an infection status may be uncertain. These types of nAbts theranostic system could also be very useful in curbing the spread of sexually transmitted infections, such as *Neisseria gonorrhea,* by applying them to contraceptives. Bacteriophages, though excluded can be classified as bio-active nanoantibiotics. These phages are virus-like particles that selectively kill bacteria when they infect them, without any damage to the eukaryotic cell [87]. Furthermore, phages can be modified with stimuli-responsive functionalities that allow for added efficacy and capabilities [88]. Additionally, non-lytic phages can be engineered to permit use as vaccines or diagnostic tools against specific bacterial infections [89].

**Fig. 6:**
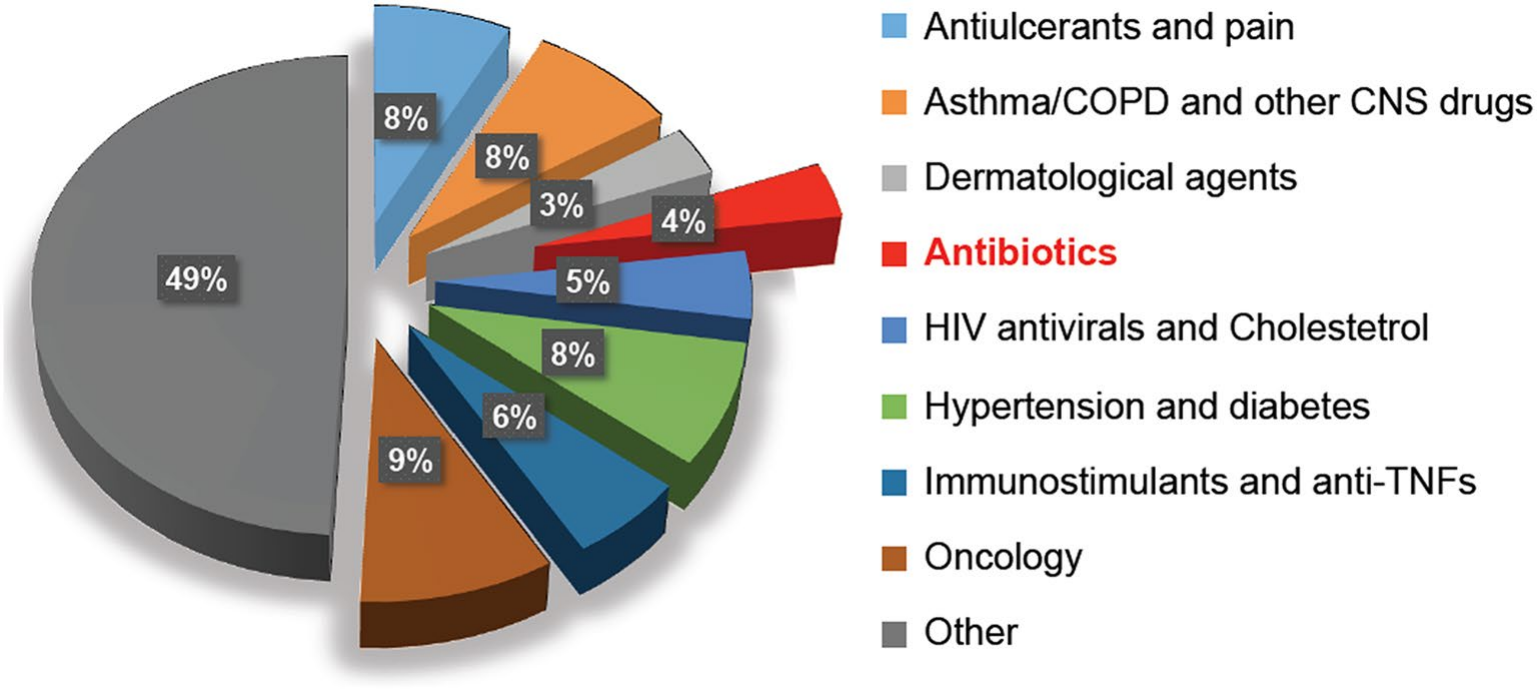
Antibiotics market share in comparison to the overall pharmaceutical market size. Antibiotics account for 4 % of the total market which is equal to or larger than some of the other markets, and has a potential for growth

It is important to note, fortunately, that the antibiotic industry is gaining some steam. Aside from the obvious need, this renewed interest in antimicrobial discovery is likely due to the expected increase in the value of the industry. BCC Research reports the market to be worth about 40.6 billion in 2015 with an expected compound annual growth rate of 2.0 % within the next 5 years [90, 91]. Regardless of the source, whether it is IBIS World Industry Report, Statista, or BCC Research Reports, there is a general agreement over the size of the current antimicrobial market. However, the forecasted direction of the market is still uncertain. This uncertainty might be caused by the upcoming patent cliffs, production of cheaper generics, or loss of efficacy due to drug-resistance formation. Irrespective of that, about 3.7 % of the over 1 trillion-dollar pharmaceuticals industry [90] is focused on antibiotics, Fig. 6. While it maybe not be the largest sector, it has been drawing increased interest. Interestingly enough, the number of clinical devices focusing on some sort of nAbts application that has received approval for use continues to increase, Table 5 [3].

**Table 5:** Examples of commercial nanoantiobiotics products

Company	Product	Composition	Current application	Development stage
Insmed	Arikace	Liposomal amikacin	Chronic *P. aeruginosa* Infection	Clinical trial (phase 3)
Staten Serum Institute	CAF09	Cationic liposome-based adjuvant	Tuberculosis, HIV	Preclinical
Smith & Nephew	Acticoat	Ag NP coated polyethylene mesh	Wound dressing	Marketed
I-Flow	ON-Q silver soaker	Ag NP coated polyvinylchloride	Catheter for delivery of local anesthetics	Marketed
Benanova	EbNP	Ag ions embedded in lignin	Nanosilver substitute	Marketed
INGMedical	Antimicrobial textiles	Electrospun textiles with metallic NPs	Medical devices	Marketed
NanoBio	NanoStat	Nanoemulsion based carrying various adjuvants	Intranasal/intramuscular vaccine delivery	Completed phase 1
IBM	–	Stimuli-responsive polymer hydrogel	Antimicrobial	Pre-clinical

## Conclusions

There is a lot of promise to be found in the field of antimicrobial therapy using nanoparticles. It is an auspicious field that might provide long-term solutions to the formation antimicrobial resistance. One of the major advantages provided by the use of the sr-nAbts system is that it allows for the bypass of traditionally antibiotic discovery pathways. The mechanism of action is not something that is already found in microbial architecture, which might cause a longer time for resistances to form. And although rapid progress is being made, there are still some topics of concern with respect to toxicity, especially to those that will manufacture the materials. With those addressed, the therapeutic possibilities are endless.
